# Rapid On-Site Sensing Aflatoxin B_1_ in Food and Feed via a Chromatographic Time-Resolved Fluoroimmunoassay

**DOI:** 10.1371/journal.pone.0123266

**Published:** 2015-04-13

**Authors:** Zhaowei Zhang, Xiaoqian Tang, Du Wang, Qi Zhang, Peiwu Li, Xiaoxia Ding

**Affiliations:** 1 Oil Crops Research Institute of the Chinese Academy of Agricultural Sciences, Wuhan 430062, P. R. China; 2 Key Laboratory of Biology and Genetic Improvement of Oil Crops, Ministry of Agriculture, Wuhan 430062, P. R. China; 3 Key Laboratory of Detection for Mycotoxins, Ministry of Agriculture, Wuhan 430062, P. R. China; 4 Laboratory of Risk Assessment for Oilseeds Products (Wuhan), Ministry of Agriculture, Wuhan 430062, P. R. China; 5 Quality Inspection and Test Center for Oilseeds Products, Ministry of Agriculture, Wuhan 430062, P. R. China; National Central University, TAIWAN

## Abstract

Aflatoxin B_1_ poses grave threats to food and feed safety due to its strong carcinogenesis and toxicity, thus requiring ultrasensitive rapid on-site determination. Herein, a portable immunosensor based on chromatographic time-resolved fluoroimmunoassay was developed for sensitive and on-site determination of aflatoxin B_1_ in food and feed samples. Chromatographic time-resolved fluoroimmunoassay offered a magnified positive signal and low signal-to-noise ratio in time-resolved mode due to the absence of noise interference caused by excitation light sources. Compared with the immunosensing performance in previous studies, this platform demonstrated a wider dynamic range of 0.2-60 μg/kg, lower limit of detection from 0.06 to 0.12 µg/kg, and considerable recovery from 80.5% to 116.7% for different food and feed sample matrices. It was found to be little cross-reactivity with other aflatoxins (B_2_, G_1_, G_2_, and M_1_). In the case of determination of aflatoxin B_1_ in peanuts, corn, soy sauce, vegetable oil, and mouse feed, excellent agreement was found when compared with aflatoxin B_1_ determination via the conversational high-performance liquid chromatography method. The chromatographic time-resolved fluoroimmunoassay affords a powerful alternative for rapid on-site determination of aflatoxin B_1_ and holds a promise for food safety in consideration of practical food safety and environmental monitoring.

## Introduction

Aflatoxin B_1_ (AFB_1_), one of the most toxic mycotoxins, is produced mainly by *Aspergillus*. It can readily contaminate both food and feed during almost all stages, e.g., before and after harvest and during storage, transportation, and consumption. It has been recognized that the fungal- or mycotoxin-induced diseases increasingly threaten both biodiversity and food security [1]. Recently, it is indicated that a fungal threat from AFB_1_ and other mycotoxins (or fungus) could cause a 39% loss in the world's food supply if severe epidemics were to occur simultaneously on all five crops [2]. Among these threats, AFB_1_ is one of the most noticeable mycotoxins due to its high oncogenicity, teratogenicity, and mutagenicity. Consequently, the study on rapid, effective, and on-site determination of AFB_1_ in the food chain has attracted tremendous efforts [3,4]. Previously, there were many determination strategies based on thin layer chromatography (TLC) or high performance liquid chromatography (HPLC) coupled with mass spectrometry (MS) [5]. However, the TLC method is negatively affected by its false-positive results and low sensitivity, while HPLC and HPLC-MS methods require valuable instruments and skilled operators and are time/labor-consuming, which are unsuitable for rapid on-site screening.

A promising trend is to use the fluoroimmunoassay (FIA) strategy with the aid of highly-sensitive and specific monoclonal (or polyclonal) antibodies. FIA, for example, enzyme-linked immunosorbent assay (ELISA), has come into analytical practice. Several studies have reported the use of this method [6]. Zhang *et al*. [7] has established an immunochromatographic assay for AFB_1_ using home-made anti-AFB_1_ mAb (3G1), producing a limit of detection (LOD) of 1 μg/kg towards agricultural products. Nevertheless, this method is suitable for qualitative detection other than quantitative one. Xu *et al*. [8] has recently developed a label-free optical sensor using gold nanorods for detecting AFB_1_, reaching a LOD of 0.16 μg/kg. However, this proposal required complex sample preparation and was suitable merely for peanut samples. In addition, an ultrasensitive determination method based on non-fouling antigen microarray has been reported by Hu *et al*. [9] using large-scale instruments, exhibiting a lower LOD of 0.003 μg/kg. However, these detection methods mentioned above are not suitable for on-site determination. Therefore, it is important to establish a rapid on-site assay for determining AFB_1_ in food and feed. Moreover, the main problem of the conventional FIA lies in strong background interference caused by complex matrices from the co-existing luminescent substances in food and feed. These interference signals mainly include scattering lights (Tyndall, Rayleigh, and Raman scattering, *etc*.) and background luminescence (optical components in the light path). Therefore, reducing background interference is essential for highly-sensitive determination.

Time-resolved fluorometry (TRF) is a noticeable alternative because the background signals with short-life (ns-μs) can be eliminated as the detection time elapses, while the fluorescent lanthanide chelates with long-life (Eu^3+^, Tb^3+^, Sm^3+^, and Dy^3+^, *etc*.) can afford an effective and reliable proportion of fluorescence signals to the contents of the sample. After the first introduction of Eu^3+^ complexes by Weissman [10], the chromatographic time-resolved fluoroimmunoassay (CTRFIA) method was found to be extremely suitable for rapid on-site assay [11]. The CTRFIA offers many advantages: (1) rapidness due to its simplicity and less time-consumption and reduced labor requirement in sample preparation and determination, (2) practicability for on-site assay due to easy automation and portability, and (3) excellent validation. The fluorescence lifetime of specific target signals is several orders of magnitude longer than that of non-specific background noises. It facilitates measurement of the lanthanide label coupled with specific targets, in which the background noise has already decayed. Therefore, the sensitivity and reliability can be enhanced dramatically.

To enhance the sensing sensitivity and reliability so as to meet the rapid on-site sensing requirement, a portable CTRFIA immunosensor was established for the determination of AFB_1_. A CTRFIA immunosensor was designed and fabricated. Base on a chromatographic time-resolved fluoroimmunoassay, results showed an enhanced sensitivity and reliability when compared with other similar immunosensors. It was found to be a considerable LOD, dynamic range, recovery, no cross-reactivity with other aflatoxins (B_2_, G_1_, G_2_, and M_1_) for food and feed matrices, such as peanuts, corn, soy sauce, vegetable oil, and mouse feed. Excellent agreement was recorded when compared with AFB_1_ determination via the HPLC method. CTRFIA immunosensor can be applied in sensing AFB_1_ in real food and feed samples, and hold promise in other food toxins determination.

## Experimental

### Chemicals and materials

All chemicals were of analytical reagent grade or higher from Sigma-Aldrich (St. Louis, MO, USA) and were used as received unless otherwise stated. AFB_1_, its antigen conjugate (AFB_1_-BSA), 1-ethyl-3-(3-dimethylamimopropy) carbodiimide (EDC), boric acid, anti-rabbit immunoglobulin, bovine serum albumin (BSA), ovalbumin (OVA), sucrose, Tween 20, methanol, and dichloromethane were purchased from Sigma-Aldrich. The 1% (solid content, W/V) Eu(III)-marked and COOH-modified monodisperse polystyrene beads were purchased from You Ni Biotechnology Co. Ltd. (Shanghai, China). Nitrocellulose membranes, sample pads, and absorbent pads were purchased from Millipore Corp. (Bedford, MA, USA). The CTRFIA buffer contains 1% sucrose, 0.5% BSA, and 2.5% Tween 20. The food and feed samples (ready-to-eat peanuts, corn, soy sauce, vegetable oil, and mouse feed) were purchased from a local supermarket (Wuhan, China). Deionic water was obtained from a Millipore Milli-Q system and used in all experiments.

### CTRFIA apparatus

**Fig 1:**
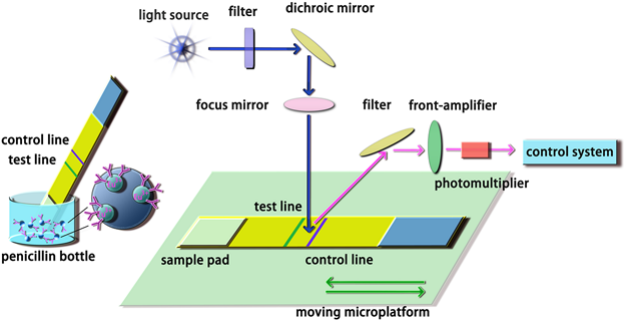
Schematic illustration of the CTRFIA sensing platform for determination of AFB_1_ in food and feed.

The CTRFIA immunosensor was shown in Fig 1. An xenon lamp was served as the excitation source. The flash lamp was activated around 1000 times at a frequency of 1 KHz by pulses from a clock-pulse generator. The emission light was acquired by a side-window photomultiplier tube at a negative bias voltage. A typical delay of 400 μs occurred when the emission light was collected from the excited light source. In this light path, the interference band-pass filters were introduced for specific excitation and emission bands. After the signals were processed using a rapid preamplifier-discriminator and pulse counter, the result was further delivered to the readout.

### Production of monoclonal antibodies against AFB_1_

The monoclonal antibody against AFB_1_ (anti-AFB_1_ mAb) was domestically produced using the hybridoma antibody technology [12]. After subcutaneous injection of antigens (AFB_1_-BSA) into a Balb/c mouse, immunization was performed four times for hybridoma selection in a two-step ELISA screening procedure. Using Balb/c mice that were treated by Freund's incomplete adjuvants, hybridoma cells were selected to prepare ascites, prior to monoclonal antibody acquisition. The resulting anti-AFB_1_ mAb was further purified by the conventional ammonium sulfate technique and stored at–20°C before use.

### Covalent coupling of Eu^3+^-microbead and anti-AFB_1_ mAb or IgG

A solution contained beads (at a final density of 1.0×1012) in boric acid buffer solution (10 mL, 0.01 M, pH 8.0) was dispersed by ultrasound for 30 s. Then, EDC solution (200 μL, 15 mg/mL) was added slowly and gently and stirred for 15 min at room temperature. After centrifugation at 150 000 g for 10 min, the precipitation was washed and centrifuged with boric acid buffer solution for twice. The beads was dissolved with boric acid buffer solution (5 mL, 0.01 M, pH 8.0) and mixed with antibody against aflatoxin B1 (300 μg) for 12 h at 4oC for conjagation. The bead-antibody mixture was centrifugated at 12 000 g for 10 min. The precipitation was dissolved with 1% BSA to close the rest of the antibody-combining sites. Finally, this precipitation was dissolved by the protective reagent containing 2.0% (w/v) BSA, 0.5% (w/v) sucrose and 0.5% (v/v) Tween-20. The resulting bead-antibody was stored at 4oC before usage. The beads were conjugated with rabbit immunoglobulin by the same manner.

### Preparation of CTRFIA strip

#### Fabrication of CTRFIA strip

An XYZ3050 Dispensing Platform, CM4000 Guillotine Cutter, and LM4000 Batch Laminator (BioDot, Irvine, CA, USA) were used to fabricate a CTRFIA strip in a modified fabrication method [7]. The sample pad was blocked with 2% (w/v) OVA to prevent nonspecific adsorption and dried at 37°C overnight, while the absorbent pad was used as received. On the nitrocellulose membrane, two adjacent lines (test line and control line) were respectively coated with AFB_1_-BSA and IgG via the BioDot XYZ3050 Platform. The distance was optimized as 5 mm between the test line and the control line to form an effective detection zone. The sample pad, nitrocellulose membrane, and absorbent pad were then assembled on a plastic scaleboard before cut into CTRFIA strips of 4 mm x 60 mm using a guillotine cutter (CM4000) and stored in a dry atmosphere at 4°C.

#### Optimization of immunoreagent concentrations on the test line and control line

The concentrations of AFB_1_-BSA and goat anti-rabbit IgG were optimized by a checkerboard method. A serial dilution from 0.05 ng/mL to 1.0 ng/mL was performed in water with a dilution factor of 2. The anti-AFB_1_ mAb-microbeads (Eu^3+^) and IgG-microbeads (Eu^3+^) were diluted as 1:50, 1:100, 1:200, and 1:400 with the protective buffer. The IC_50_ values were recorded for acquiring optimum concentrations.

#### Optimization of reaction conditions

The reaction temperature was 37°C. In order to obtain the optimal reaction volume, a blank peanut matrix spiked with 5 μg/kg AFB_1_ was employed, and a series of reaction volumes from 50 μL to 500 μL were tested via the standard protocol. To acquire the most possible minimum incubation time, tests were conducted on a blank peanut matrix spiked with 5 μg/kg AFB_1_ using different incubation time from 2 min to 15 min.

### Evaluation of the CTRFIA immunosensor

Various AFs-free matrices determined by HPLC were employed as blank matrices to evaluate the performance parameter of CTRFIA immunosensor. Each experiment was carried out successively in triplicate to record the mean readout value.

#### Standard curves

Standard curves depend on various blank matrices spiked by AFB_1_ at a series of concentrations. For example, as to peanuts, blank peanut samples were spiked with AFB_1_ of 0.1, 0.5, 1.0, 5.0, 10.0, 15.0, and 20.0 μg/kg, respectively. Using the CTRFIA immunosensor, the fluorescent intensities obtained from the test line and control line were recorded as the value (T) and value (C), respectively. The ratio of the value (T) to the value (C) was read as the signal value (Y) or the value (T/C). A standard curve was a fitted function expressed as the signal value versus the natural logarithm of AFB_1_ concentration (X) in the equation: Y (signal value) = b X (ln *c*_AFB1_) + a. The sensitivity was defined as the slope of the standard curve according to previous reports [13,14].

#### Linear range

The linear range started from the limit of qualification (LOQ) and ended at the cut-off concentration where the color faded or the linear range was exceeded. The detection linear range was investigated by using ready-to-eat peanut samples spiked with AFB_1_ at a series of concentrations from 0 to 50 μg/kg. For other samples, the same manner was employed.

#### Recovery

A standard recovery towards AFB_1_ was recorded to evaluate the CTRFIA accuracy in food and feed samples. After determined as AFB_1_-free samples via an HPLC method, AFB_1_-free food and feed samples were spiked with AFB_1_ standard solution to achieve concentration gradient series. These serial dilutions containing AFB_1_ from 0.06 μg/kg to 60 μg/kg were spiked into AFB_1_-free samples before determination by CTRFIA sensing with each assay repeated 3 times.

#### Precision

Inner- and inter-day sensing precision with AFB_1_-spiked samples was studied using a CTRFIA strip with the assay repeated three times. The inter-day precision was conducted for 30 successive days.

#### Specificity

Peanut as a representative sample, specificity was investigated by using cross-reactivities of CTRFIA strip with aflatoxin B_2_, G_1_, G_2_ and M_1_ at the concentration of 20 μg/kg, according to previous report [15].

### Sample preparation

Naturally-contaminated food samples (ready-to-eat peanuts, corn, soy sauce, vegetable oil, and mouse feed) were collected from different local markets, which were analyzed by the CTRFIA method and further confirmed using HPLC. The sample preparation depending on the sample matrices is described in the following sections.

#### Solid sample preparation for CTRFIA

Solid samples, such as ready-to-eat peanuts, corn, and mouse feed, were finely ground using a commercialized homogenizer (Jiuyang soybean milk machine, China). Briefly, 20 g solid sample was dissolved in methanol/water (7:3 v/v) and ground for 2 min prior to filtration, 1 mL filtrate was diluted with 3 mL CTRFIA buffer, and then the dilution was filtered with 0.45 μm filter membrane before CTRFIA sensing.

#### Liquid sample preparation for CTRFIA

For vegetable oil, 5 g sample was extracted by 15 mL methanol/water (7:3 v/v) containing 1% NaCl. The resulting separated 1 mL methanol/water layer was diluted by 3 mL CTRFIA buffer before filtered by 0.45 μm filter membrane.

For soy sauce, 2 g sample was extracted using a mixture of 4 mL water and 2 mL dichloromethane. The separated 1 mL dichloromethane layer was volatilized and further re-dissolved with 1 mL methanol and 2 mL CTRFIA buffer. This re-dissolved mixture was filtered with 0.45 μm filter membrane before use.

#### Sample preparation for HPLC

Sample extraction followed the above procedure. After filtration, 4 mL extracting solution was extracted by 2 mL petroleum ether thoroughly. Then, 8 mL water was added to the 3 mL separated methanol/water layer and the mixture was filtered via 0.45 μm filter membrane. A home-made immunoaffinity column loaded with anti-AFB_1_ mAb was conducted to enrich and purify AFB_1_ from the filtrate. The resulting elution containing AFB_1_ was further derived with trifluoroacetic acid before HPLC determination.

### Determination of AFB_1_ in food and feed

A penicillin bottle with IgG-microbead (Eu^3+^) and anti-AFB_1_ mAb-microbead (Eu^3+^) were vacuum freeze-drying preserved, using BSA, Tween 20 and glucose as stabilizer. After the samples were prepared depending on their matrices, 300 μL sample dilution was transferred into this penicillin bottle. Free AFB_1_ and anti-AFB_1_ mAb-microbead (Eu^3+^) were mixed in a penicillin bottle and laterally flowed through the CTRFIA strip via capillary action. After specific recognition, the results could be read out in 6 min using the proposed CTRFIA platform.

### CTRFIA validation in comparison with HPLC

The randomly-selected food and feed samples were prepared and determined simultaneously by CTRFIA and HPLC for comparison.

## Results and discussion

### Basic interpretation of CTRFIA sensing

**Fig 2:**
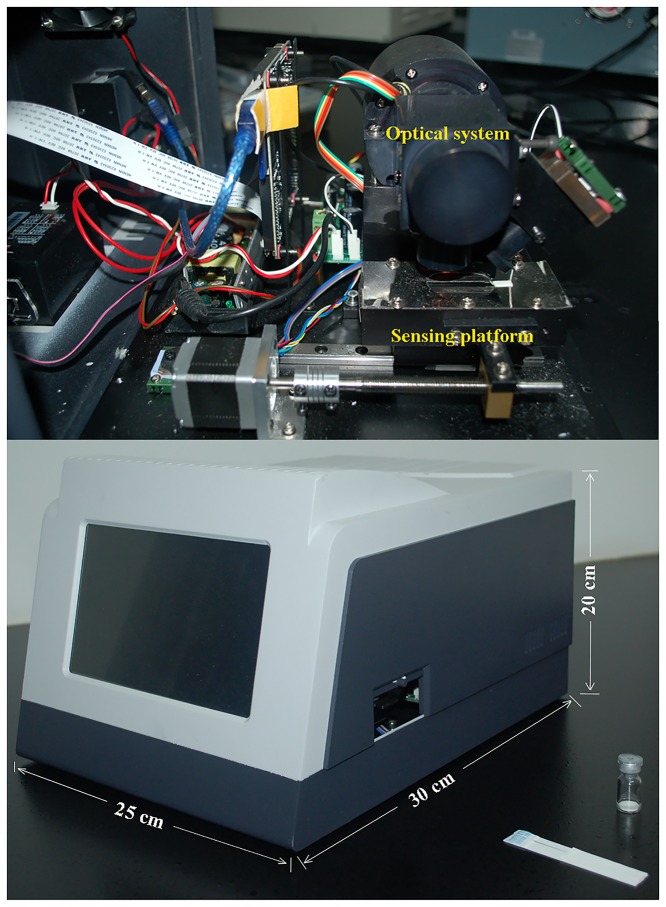
The external and inner view of CTRFIA sensor.

Based on the home-made CTRFIA sensor (Fig 2), a rapid on-site immunoassay with enhanced sensitivity was developed for determination of AFB_1_ in food and feed. This CTRFIA sensor consisted of four parts, that is, an optical system with a lamp and PMT, a sensing motion platform with a controlled motor, a controlling circuit, and an output date interface. The size of this immunosensor was 30 cm×25 cm×20 cm, for length, width, and height, respectively. Its weight was about 0.8 kg, suggesting a convenient portable device. Moreover, it can be powered by residential electricity (220 V), vehicle power supply (12V, 5A, 65W), or portable power source (12V, 36Ah/10HR), with aid of a variable pressure charging system (output: 12V, 3.34A, 40W). A variable pressure charging system allowed its portability and availability. It has been generally recognized that Eu^3+^ complexes have advantages over conventional fluorescent compounds. Firstly, the emission lifetime of the Eu^3+^ chelate is longer. Secondly, it has large Stokes shifts and can emit visible fluorescence. Thirdly, its emission peak width at half height is as narrow as 10 nm. The inherent merits provide an effective means in the detection of AFB_1_.

During AFB_1_ detection, a penicillin bottle was employed to reserve IgG-microbead (Eu^3+^) and anti-AFB_1_ mAb-microbead (Eu^3+^) in order to guarantee the stability of sensing reagents and reduce the inter-batch error. The extracted sample dilution was added to this penicillin bottle pre-embedded with anti-AFB_1_ mAb-microbeads (Eu^3+^) and IgG-microbeads (Eu^3+^) before typical competitive immunochromatographic assay. The CTRFIA strip was then put into the CTRFIA immunosensor. The optical devices were fixed, while the CTRFIA strip could be translated using a moving platform in order to realize CTRFIA sensing. The test line and control line were visible under ultraviolet light other than visible lights. The fluorescence intensity was recorded by the readout, indicating a negative correlation with the AFB_1_ concentration.

### Preparation of anti-AFB_1_ mAb-microbead (Eu^3+^) and IgG-microbead (Eu^3+^)

The fluorescence spectra of the microbeads before and after conjugation with anti-AFB_1_ mAb or IgG were recorded around 617 nm via a fluorescence detector. The reduced fluorescence intensity after the conjugation with anti-AFB_1_ mAb or IgG proved the coupling of microbeads with anti-AFB_1_ mAb or IgG. In addition, the TEM images of the microbeads could help to confirm the conjugated microbeads.

### Sample preparation

A major challenge in aflatoxin determination lies in extremely-low AFB_1_ concentrations found in food and feed samples. To address this challenge, proper sample preparation with a wide application range is required. In order to develop a widely applicable sample preparation method, food and feed samples were divided into solid and liquid sample groups. Several typical food and feed samples (ready-to-eat peanuts, corn, soy sauce, vegetable oil, and mouse feed) were used as model samples. The proposed sample preparation technique was suitable for a rapid screening method.

A one-step sample preparation procedure involving grinding and extraction was proposed for solid samples. This improved one-step method could efficiently extract AFB_1_ from solid samples within 2 min, dramatically reducing the operating time and labor and being suitable for rapid on-site immunoassay. A simple liquid-liquid extraction method was used for AFB_1_ extraction from liquid samples. As for soy sauce, the pigment and high-concentration salt negatively affected accurate CTRFIA determination by disturbing the anti-AFB_1_ mAb activity and fluorescence intensity. The employment of dichloromethane during extraction, followed by volatilization of dichloromethane and re-dissolving with methane, could effectively remove the original high-concentration salt and pigment.

Methanol, acetone, and acetonitrile were tested for extraction as organic solvents, among which the highest CTRFIA sensitivity was found using methanol. Various methanol concentrations from 50% to 90% were further tested for assessing extraction efficiency. With more methanol contents, the extraction efficiency was enhanced. However, a high methanol concentration could hamper the activity of anti-AFB_1_ mAb on a CTRFIA strip. For some oil-rich samples such as vegetable oil and mouse feed, the addition of 1% sodium chloride benefited the demulsification process. Considering these two aspects, 70% methanol was chosen for the grinding and extraction solution.

After grinding and extraction, the filtered extract was further diluted by the CTRFIA buffer to reduce the AFB_1_ concentration and obtain a stable result. The dilution factor of the extract was optimized from 1:1 to 1:5. A lower dilution factor allowed unstable CTRFIA results possibly because methanol at a higher concentration inactivated anti-AFB_1_ mAb and was inefficiently mixed with a microbead (Eu^3+^) probe in a penicillin bottle. However, while decreasing the matrix effect and affording stable results, a higher dilution factor lowered the LOD, especially in consideration of soy sauce whose maximum residue level (MRL) for AFB_1_ is set in China as 5 μg/kg. Taking these two factors into consideration, a dilution factor was optimized as 1:3.

Before incubation with a CTRFIA strip at 37°C followed by determination via the immunosensor, the dilution was filtered to remove impurities. Using simplified sample preparation, it is possible to realize a rapid on-site determination of AFB_1_.

### Optimization of the CTRFIA strip

The immunoreagent concentrations on the test line and control line, as well as reaction conditions were optimized for high sensitivity and specificity.

The immunoreagent concentrations were optimized by checkerboard titration for minimum immunoreagent consumption, which guaranteed a clear red-line on the test line for negative samples. It was found that an optimized probe with 6 μg/mL anti-AFB_1_ mAb microbead (Eu^3+^) allowed a sensitive determination of AFB_1_ in food and feed. In addition, 0.6 μL/cm AFB_1_-BSA (0.5 mg/mL) and 0.4 μL/cm IgG (0.5 mg/mL) were optimized to dispense on the test line and control line, respectively.

The immune reaction was performed at 37°C as previously reported, with the reaction volume of 300 μL and reduced reaction time of 6 min selected for the CTRFIA.

### Evaluation of the CTRFIA sensing

**Fig 3:**
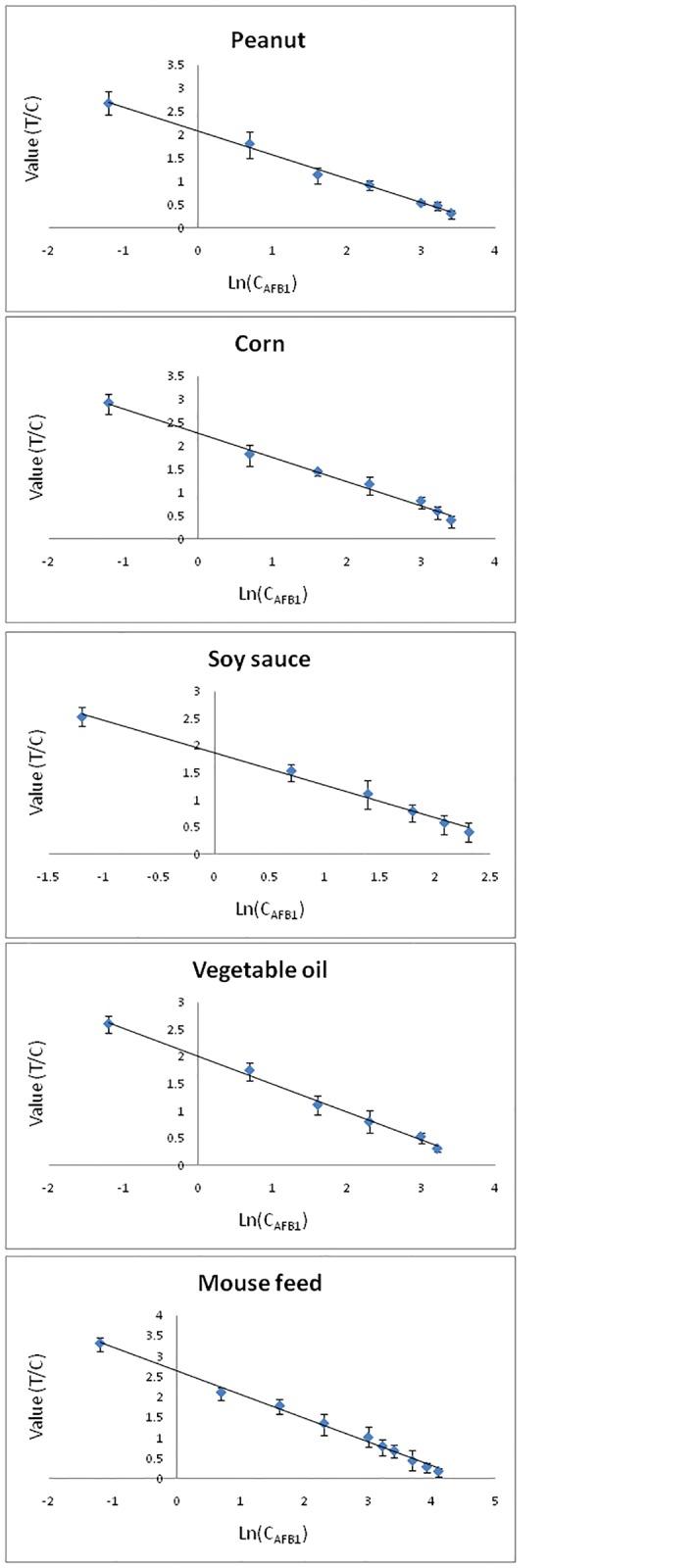
Standard curves for AFB_1_ detection using CTRFIA.

Several food and feed samples (peanuts, corn, soy sauce, vegetable oil, and mouse feed) were utilized to evaluate the proposed CTRFIA immunosensor. The linear range of standard curves was determined by plotting the ratio of the test value to the control value (Y, signal value) versus the natural logarithm of concentrations (ln *c*), expressed by the equation: Y = b·ln[*c*_*AFB1*_] + a, depending on different matrices. It was summarized in Table 1 and Fig 3 containing the standard curve, LOD, LOQ, together with the linear range.

**Table 1:** CTRFIA determination in peanuts, corn, soy sauce, vegetable oil, and mouse feed.

Sample	SD of 21measurements in blank matrices	Standard curve	LOD(μg/kg)	LOQ(μg/kg)	Linear range(μg/kg)	R^2^
Peanut	0.0153	Y = -0.511X + 2.080	0.09	0.3	0.3–30	0.995
Corn	0.0104	Y = -0.516X + 2.274	0.06	0.2	0.2–30	0.991
Soy sauce	0.0181	Y = -0.598X + 1.869	0.09	0.3	0.3–10	0.9921
Vegetable oil	0.0156	Y = -0.512X + 2.011	0.09	0.3	0.3–25	0.9907
Mouse feed	0.0234	Y = -0.579X + 2.636	0.12	0.4	0.4–60	0.9904

Taking into account of the blank matrices and signal noises, the LOD and LOQ were calculated as recommended by International Union of Pure and Applied Chemistry (IUPAC) [13]. After the standard deviations (SDs) were obtained from 21 measurements of AFs-free matrices of different samples, each LOD was calculated as a triple of the SD divided by the absolute slope of the standard curve. The LOQ was calculated as around 3.3 times the LOD, representing the lower limit of the linear range. The upper limit of the linear range depended on the color-fading of the test line. The similar LODs from 0.06 μg/kg to 0.09 μg/kg corresponding to the low LOQs of 0.3 μg/kg suggested the reduced matrix effect among various food and feed after sample preparation. The LODs were dramatically lower than that in the previous study by Zhang *et al*. (1 μg/kg) [12] and other optical biosensors using gold nanorods (0.16 μg/kg) [8], probably due to both the higher density of anti-AFB_1_ mAb conjugated with microbeads (Eu^3+^) and the delayed detection that offered the absence of autofluorescence with CTRFIA. As in Table 2, in comparison with other optical immunosensors for AFB_1_ sensing, e.g., quantum dot-based FRET sensor [16], microfluidic strip sensor [17], optical waveguide light-mode spectroscopy based sensor [18], magnetic based fluorescence immunoassay [19], and SPR sensor [20], *etc*., the as-established CTRFIA sensor exhibited a comparative or higher sensitivity. More importantly, the sensor previously reported allowed only the determination of single sample such as peanut, rice, corn, while the CTRFIA sensor provided dramatic wider linear range and the wide applied range in both food and feed.

**Table 2:** Comparison of CTRFIA method with other reported sensing method for AFB_1_ sensing.

Method	Samples	LOD(μg/kg)	Linear range(μg/kg)	Reference
CTRFIA	Peanut	0.09	0.3–30	This work
	Corn	0.06	0.2–30	
	Soy sauce	0.09	0.3–10	
	Vegetable oil	0.09	0.3–25	
	Mouse feed	0.12	0.4–60	
A homogeneous immunosensor based on FRET between different-sized quantum dots	rice	0.04	0.06–5	[16]
Microfluidic Smectite-Polymer Nanocomposite Strip Sensor	corn	sub-100	20-80/5-15	[17]
Immunosensor based on OWLS technique	grain	<0.5	0.5–10	[18]
Magnetic Mesoporous silica nanocontainers based fluorescence immunoassay	Peanut	0.008	0.01–5	[19]
A bifunctional protein crosslinker-based surface plasmon resonance biosensor	corn	1000	NA	[20]

The linear range, wider than the previous report (0.5–20 μg/kg) [8], depended on different matrices (salinity, pH value, background color, fat content, *etc*.). The linear range met the MRLs set by both EU and China, suggesting that CTRFIA sensing could be employed as an effective rapid on-site sensing method. For example, the linearity toward peanuts ranged from 0.30 μg/kg to 30.00 μg/kg, satisfying the MRLs set by both EU (2 μg/kg) and China (20 μg/kg), while that toward soy sauce of 0.30–10.00 μg/kg fulfilled the MRL set in China (5 μg/kg).

Recoveries from various matrices were evaluated by three concentration levels, such as the concentration levels around LOD and MRL and an optional level (Table 3). The results indicated considerable recoveries from 80.5% to 116.7% with an average of 100.8%, due to the widely-applicable sample preparation that eliminated the matrix effect.

**Table 3:** Recoveries of CTRFIA sensing via spiked blank matrices (n = 3).

Sample numbers		Spiked AFB_1_ (μg/kg)	Found AFB_1_ (μg/kg)	Recovery (%)
peanut	1	0.09	0.09±0.0075	100.0
	2	2	2.1±0.14	105.0
	3	20	21.2±1.1	106.0
corn	1	0.06	0.07±0.0055	116.7
	2	10	9.3±0.6	93.0
	3	20	16.7±0.8	83.5
soy sauce	1	0.09	0.1±0.008	111.1
	2	2	2.1±0.14	105.0
	3	5	4.4±0.36	88.0
vegetable oil	1	0.09	0.09±0.0078	100.0
	2	5	4.8±0.35	96.0
	3	10	10.5±0.6	105.0
mouse feed	1	0.12	0.14±0.012	116.7
	2	20	18.4±1.2	92.0
	3	50	47.1±2.5	94.2

The intra- and inter-day precision coefficients of variation (CVs) were determined from 3 measurements and 30 days, respectively. The low and high levels of AFB_1_ spiked samples were conducted in precision determination. As shown in Table 4, the average inter-day CVs were between 3.1% and 6.1%, while the intra-day CVs were recorded from 6.1% to 8.9%. The resulting excellent precision and stability satisfied the requirement for CTRFIA sensing.

**Table 4:** Intra- and inter-day CV tests of CTRFIA sensing.

Sample	Spiked AFB_1_(μg/kg)	Intra-day (n = 3)		Inter-day (30 days n = 3)	
			
		Found (μg/kg)	CV (%)	Average found(μg/kg)	Average CV(%)
Peanut	2	1.9	6.2	2.1	7.2
	20	20.1	6.5	20.0	6.1
Corn	2	1.8	4.8	2.0	7.5
	20	20.0	5.6	20.0	7.1
Soy sauce	1	0.91	5.2	1.03	7.5
	5	4.8	4.5	4.9	6.2
Vegetable oil	10	10.2	4.7	10.5	7.9
	20	20.1	4.8	20.4	6.8
Mouse feed	10	9.4	5.1	10.5	6.8
	50	47.8	7.1	50.5	8.9

**Fig 4:**
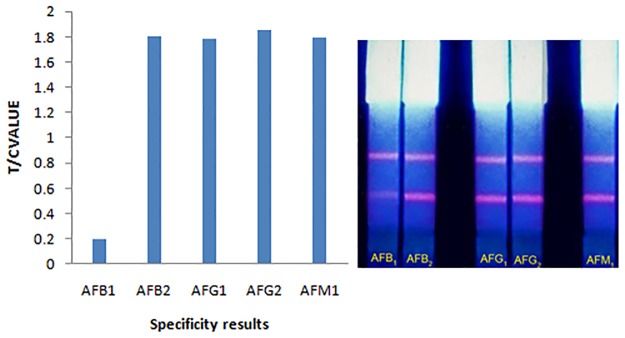
Cross-reactivities of AFB_1_ CTRFIA strip with AFB_2_, G_1_, G_2_ and M_1_ at the concentration of 20 ng/mL.

Specificity results demonstrated no inhibition to color intensity for 20 μg/kg of aflatoxin B_2_, G_1_, G_2_, respectively, compared with aflatoxin B_1_ negative control (Fig 4). This could be attributed to the high selectivity of monoclonal antibody against aflatoxin B_1_.

### Determination of AFB_1_ in food and feed

The practicability of CTRFIA sensing in food and feed was evaluated under the optimal conditions. Five sample branches, namely, peanuts, corn, soy sauce, vegetable oil, and mouse feed, were purchased in local markets. Each sample branch consisted of 5 different samples, and each sample was measured three times. The CTRFIA was validated by comparing the AFB_1_ concentrations by HPLC. The uncontaminated samples (AFs-free) were served as negative control samples. After CTRFIA and traditional HPLC were compared, the results via CTRFIA allowed an excellent agreement with those by HPLC (Table 5). The relative standard deviations (RSDs) from these food and feed samples were below 10%, and the recoveries ranged from 83.8% to 106.1%, meeting sensing requirements. The determination of AFB_1_ in real food and feed sample suggested that CTRFIA could serve as an effective and reliable sensing alternative in rapid on-site assay.

**Table 5:** Comparison of CTRFIA and HPLC methods for AFB_1_ in food and feed samples.

Sample	No.	Result by HPLC (μg/kg)	Results by CTRFIA (μg/kg)				RSD(%)	Recover(%)
			1	2	3	Mean		
Peanut	1	5.2	4.7	5.4	5.3	5.1	7.4	98.7
	2	1.2	1.0	1.0	1.1	1.0	5.6	86.1
	3	7.6	7.2	7.3	7.9	7.5	5.1	98.2
	4	15.5	15.5	17.1	16.9	16.5	5.3	106.5
	5	0.8	0.7	0.7	0.8	0.7	7.9	91.7
Corn	1	0.6	0.7	0.6	0.6	0.6	9.1	105.6
	2	1.1	1.0	1.1	1.0	1.0	5.6	93.9
	3	5.2	5.2	5.8	5.6	5.5	5.5	106.4
	4	9.1	9.6	9.0	9.5	9.4	3.4	102.9
	5	7.5	7.5	7.9	8.1	7.8	3.9	104.4
Soy sauce	1	n.d.	n.d.	n.d.	n.d.	n.d.	-	-
	2	0.7	0.7	0.6	0.7	0.7	8.7	95.2
	3	2.5	2.4	2.5	2.5	2.5	2.3	98.7
	4	3.8	3.8	3.4	3.8	3.7	6.3	96.5
	5	4.7	4.7	4.4	4.8	4.6	4.5	98.6
Vegetable oil	1	n.d.	n.d.	n.d.	n.d.	n.d.	-	-
	2	1.5	1.5	1.5	1.4	1.5	3.9	97.8
	3	5.4	5.0	5.3	5.5	5.3	4.8	97.5
	4	8.4	8.4	9.0	8.6	8.7	3.5	103.2
	5	12.2	12.0	13.4	12.3	12.6	5.9	103.0
Mouse feed	1	8.8	9.0	9.1	8.8	9.0	1.7	101.9
	2	5.9	5.9	6.2	6.0	6.0	2.5	102.3
	3	19.7	20.5	20.4	21.3	20.7	2.4	105.2
	4	25.4	26.3	26.1	24.5	25.6	3.8	100.9
	5	42.3	43.2	44.0	42.4	43.2	1.9	102.1

n.d. is short for "not detected."

## Conclusion

A portable rapid on-site immunosensor was developed based on CTRFIA strips for the determination of AFB_1_ in real food and feed samples. A universal sample preparation method was proposed for solid and liquid samples so as to allow practical applications. The LODs of 0.09, 0.06, 0.09, 0.09, and 0.12 μg/kg were found for peanuts, corn, soy sauce, vegetable oil, and mouse feed, respectively. A lower LOD and wider linear range that depended on different matrices satisfied the strict MRLs set by EU and China. The ultra-sensitivity and reliability of the method could be attributed to the delayed time-resolved fluorescence which eliminated the background noise caused by autofluorescence and other scattered lights. Considerable recoveries were found to be from 80.5% to 116.7% for all matrices. The intra- and inter-day measurements offered CVs lower than 10%, fulfilling daily application requirements. Compared with that via HPLC, the results from CTRFIA allowed an excellent agreement. This proposed CTRFIA sensing method can be used as a promising strategy for sensitive, rapid, on-site determination of AFB_1_ in food and feed samples.
